# Chitosan Mitigates Functional Deterioration of Myofibrillar Protein After Chlorogenic Acid-Induced Oxidation: Structure Restoration and Interfacial Regulation

**DOI:** 10.3390/foods15142420

**Published:** 2026-07-08

**Authors:** Junren Zhao, Yugang Ji, Wenjing Tao, Chun Wang, Zhimei Tang, Yujia Shi, Huiyun Zhang

**Affiliations:** 1 School of Biology and Food Engineering, Guangdong University of Petrochemical Technology, Maoming 525000, China; zhaojunren@gdupt.edu.cn (J.Z.);; 2 Guangdong Provincial Key Laboratory for Green Agricultural Production and Intelligent Equipment, Maoming 525000, China; 3 School of Food and Bioengineering, Henan University of Science and Technology, Luoyang 471003, China

**Keywords:** myofibrillar protein, chlorogenic acid, chitosan, emulsifying properties, gel quality

## Abstract

Chlorogenic acid (CA) exhibits robust lipid antioxidant activity within meat matrices. However, excess CA generates quinones that alter and damage porcine myofibrillar protein (MP). This study investigated the restorative effects of chitosan (CS) on MPs suffering from CA-induced oxidative damage. Three CA concentrations (0, 50, 100 μmol/g protein) and five CS dosages (0.125–1.0 g/g protein) were used to evaluate conformation, turbidity, surface hydrophobicity, solubility, emulsification, rheology, and gel properties. CA-oxidative damage to MP triggered protein unfolding, thiol depletion and aggregation, greatly lowering solubility, emulsifying capacity, viscoelasticity and water retention. CS exerted biphasic effects on turbidity, surface hydrophobicity, tertiary structure, and solubility only under severe CA-induced oxidative modification (100 μmol/g CA): low-to-medium CS aggravated adverse changes, while 1.0 g/g CS partially reversed such damage. For conformational, emulsion and gel parameters, CS consistently alleviated structural disorder caused by CA-induced oxidative damage across all treatments, with 1.0 g/g CS optimally mitigating α-helix loss and uneven emulsion droplets. Significant CA × CS interactions were detected for conformation, turbidity, surface hydrophobicity, solubility, emulsification and rheology (*p* < 0.001). Gel strength, water-holding capacity and water distribution exhibited non-significant interactions (*p* > 0.05), revealing independent additive effects of CA and CS on gel networks. Overall, high-dose CS partially ameliorates structural and functional defects of MP caused by CA-induced oxidative damage, which provides theoretical support for the combined application of polyphenols and polysaccharides in meat protein regulation.

## 1. Introduction

Myofibrillar proteins (MPs) act as the primary structural and functional components of muscle tissue. Their abilities to form gels, stabilize emulsions, and retain water significantly influence the texture, moisture retention, and overall sensory quality of meat-based foods [1]. However, MPs are highly vulnerable to free radical-induced oxidation during processing, storage, and transportation. Such radical-driven structural rearrangements trigger the unfolding, aggregation, and subsequent impairment of their functional properties [2]. To mitigate this issue, natural polyphenols have emerged as promising antioxidants in meat processing because of their safety and strong free-radical scavenging activity. Chlorogenic acid (CA), a widely distributed hydroxycinnamic acid-derived polyphenol found in coffee beans, fruits, and vegetables, has been well-documented for its ability to inhibit lipid oxidation in meat products [3,4].

Despite their antioxidant advantages, natural phenolics typically need higher concentrations to attain an efficacy comparable to that of synthetic antioxidants [5]. Significantly, excessive doses of phenolics can have dual adverse impacts on MP functionality. While inhibiting lipid oxidation, they may promote protein oxidation and disrupt gel formation. For example, high concentrations of CA can react with the thiol groups of MP to form thiol–quinone adducts, which impede the development of disulfide bridges during thermal gelation [6]. This process is accompanied by the depletion of thiol groups, an increased exposure of hydrophobic domains, the destabilization of MP secondary and tertiary structures, and the accumulation of protein carbonyls. Ultimately, it results in protein aggregation, a reduced ability to retain water, a decreased gel firmness, and an impaired emulsion stability [7,8]. Specifically, Cao and Xiong [9] reported that when porcine MP is exposed to 150 μmol of CA per gram of protein, it results in significant alterations in its α-helical conformation and overall tertiary structure. Consequently, this impairs the gel strength and water-holding capacity (WHC) of the protein. Likewise, structurally related polyphenols like epigallocatechin gallate at 50–100 μmol/g protein induced comparable damage in pork MP, including thiol loss, elevated hydrophobicity, and diminished gel strength [10]. CA concentrations of 50 and 100 μmol/g protein were selected to simulate routine moderate polyphenol supplementation and excessive addition commonly tested during the development of functional meat products. This selection highlights the necessity of balancing the lipid antioxidant benefits of high-dose CA with the intact MP structure, gel performance, and emulsion stability in meat manufacture. Although numerous studies have clarified the changes in the structure and gel properties of MP induced by phenolics, research that focuses on targeted strategies to alleviate the multidimensional deterioration caused by CA is still relatively limited, especially with respect to the regulatory function of cationic polysaccharides.

Chitosan (CS), a cationic polysaccharide that is derived from the partial or complete deacetylation of chitin and whose solubility and surface charge density are strongly pH-dependent, is composed of repeating units of D-glucosamine and N-acetyl-D-glucosamine linked by β-(1→4) glycosidic bonds [11]. At the experimental pH of 6.0 in our study, the partial deprotonation of amino groups reduces its net positive charge and tunes the electrostatic attraction with negatively charged quinone-modified MP. Due to its outstanding biocompatibility, biodegradability, thickening, stabilizing, and rheological behavior, CS has found widespread application in food processing and product development [12]. Previous studies have demonstrated that CS can bind to meat proteins via multiple non-covalent mechanisms, namely electrostatic forces, hydrogen bonding, and hydrophobic interactions, which in turn modulate the functional behavior of meat proteins. For example, CS has been demonstrated to significantly enhance the WHC and gel firmness of MP gels by forming a synergistic polysaccharide–protein network [12]. It also improves the thermal stability and storage modulus of salt-soluble meat protein gels through electrostatic binding [13]. Furthermore, CS stabilizes oil-in-water emulsions, both by enhancing the viscosity of the continuous phase and by strengthening interfacial film formation [14]. Notably, CS exhibits antioxidant activity in protein systems, effectively inhibiting protein oxidation through metal ion chelation and reactive oxygen species scavenging [15]. These distinctive multi-functional properties indicate that CS has the potential to serve as a modulator to alleviate the adverse impacts of high-dose CA on MP.

Although individual effects of CA or CS on MP properties have been reported in the previous literature, the interactive regulation mechanism and dose-dependent alleviation effect of CS on CA-induced structural deterioration, emulsion instability and gel network damage of MP remain poorly understood. Therefore, this research comprehensively investigated how different concentrations of CS affect the structural characteristics, emulsifying capacity, rheological properties, gel-forming ability, WHC, and spatial distribution of water within MP under two CA modification levels, specifically 50 and 100 μmol/g protein. The objectives were to clarify potential regulatory pathways through which CS may alleviate CA-induced structural and functional damage to MP, and to offer a theoretical foundation for the collaborative application of CA and CS in the quality regulation of muscle protein-based food products.

## 2. Materials and Methods

### 2.1. Materials

Porcine longissimus dorsi muscle was sourced from a local commercial slaughterhouse (freshly slaughtered within 2 h) and delivered to the lab on ice within 30 min. Soybean oil (food grade) was obtained from a local supermarket. Chlorogenic acid (CA, ≥98% purity), chitosan (CS, deacetylation degree ≥ 85%, molecular weight 50 kDa), bromophenol blue, 5,5′-dithiobis-(2-nitrobenzoic acid) (DTNB) were purchased from Aladdin Reagent Co., Ltd. (Shanghai, China). Other analytical-grade reagents were obtained from Sinopharm Chemical Reagent Co., Ltd. (Shanghai, China). All other reagents used were of analytical grade unless otherwise specified.

### 2.2. Extraction of Myofibrillar Protein

Myofibrillar proteins were extracted from porcine longissimus dorsi muscle at 4 °C following an earlier published protocol with minor adjustments [8]. In brief, 150 g of minced skeletal muscle tissue was manually trimmed to remove visible adipose and connective tissues, then homogenized in 600 mL of ice-cold extraction buffer (comprising 0.1 M NaCl, 2 mM MgCl_2_, 1 mM EDTA, and 0.1 M phosphate buffer, pH 7.2) using a high-speed rotor-stator homogenizer at 20,000 rpm for 3 min on ice. The homogenate was centrifuged at 4500× *g* for 15 min at 4 °C, and the resulting pellet (containing MP) was subjected to the same extraction process twice more to remove sarcoplasmic proteins. The protein sediment was then washed three times with a 0.1 M sodium chloride solution under identical centrifugation parameters. The final MP pellets were resuspended in 0.1 M NaCl solution and kept at 4 °C prior to use. Protein concentration was quantified using the Biuret assay, with bovine serum albumin (BSA) as the calibration standard; the mean protein content was 93.87 ± 3.62 mg/g.

### 2.3. Fabrication of MP–CA–CS Ternary Systems

Freshly extracted MP was dispersed in ice-cold 0.1 mol/L NaCl solution under gentle magnetic stirring at 4 °C to yield a final MP concentration of 40 mg/mL. CA was pre-dissolved in absolute ethanol (10%, *v*/*v*) to avoid aggregation, then added dropwise to the MP suspensions to achieve target concentrations of 50 or 100 μmol/g (based on total MP content). The two CA concentrations were selected based on previous functional meat studies and realistic product development scenarios, representing routine moderate polyphenol antioxidant supplementation and excessive overdosage that induces MP quality deterioration, respectively [9,10]. The resulting MP–CA binary mixtures were continuously stirred at 4 °C for 30 min to allow sufficient protein oxidation and thiol–quinone adduction prior to CS addition.

Subsequently, CS was pre-dissolved in a 1% (*v*/*v*) aqueous solution of glacial acetic acid at ambient temperature with continuous magnetic stirring for 2 h to prepare a clear 2% (*w*/*v*) stock solution (pH = 4.0–4.5), ensuring complete dissolution without flocculation. This acidic CS stock solution was gradually added dropwise to the MP-CA mixtures with continuous gentle stirring to attain target CS concentrations of 0.125, 0.25, 0.5, and 1.0 g/g protein. The CS concentration was determined according to preliminary experiments and published polysaccharide–meat protein interaction literature [13,16]. After the addition of CS, the pH of the whole ternary system was slowly adjusted to 6.0 with 0.1 mol/L NaOH. CS is a pH-dependent cationic polysaccharide with solubility and surface charge density highly sensitive to pH values. CS carries maximum positive charge under acidic dissolution conditions (pH 4.0–4.5) for full solvation. After adjustment to the experimental pH of 6.0 (close to physiological meat pH), partial deprotonation of CS amino groups reduces its net positive charge, which moderately modulates the electrostatic attraction between cationic CS and negatively charged quinone-modified MP, thereby dominating the non-covalent interaction and regulatory behavior in the MP–CA–CS ternary system. The final volume fraction of glacial acetic acid in the ternary system was controlled below 0.1% (*v*/*v*), which had no negative impacts on the structural and functional performance of MP.

The MP–CA–CS ternary mixtures were then stored at 4 °C for 12 h with gentle orbital shaking (60 rpm) to promote sufficient non-covalent binding among the components. Three distinct reference systems were established for comparative analysis: (1) blank control (MP suspended in 0.1 mol/L NaCl with equivalent acetic acid and NaOH as used in CS dissolution and neutralization, without any additives, pH adjusted to 6.0); (2) negative control (MP samples treated solely with 50 or 100 μmol/g of CA, with equivalent acetic acid and NaOH, without incorporation of CS; pH adjusted to 6.0); and (3) chitosan-only control series: MP samples containing graded CS concentrations (0.125–1.0 g/g protein) without CA, with corresponding acetic acid and NaOH treatments, and the pH adjusted to 6.0, were used to isolate the intrinsic effect of CS on unoxidized MP.

### 2.4. Structural Characterisation of MP Samples

#### 2.4.1. Turbidity Assessment

The turbidity of MP samples (0.4 mg/mL) was measured at 25 °C using a UV-visible spectrophotometer (U-5100, Hitachi, Tokyo, Japan) by monitoring the absorbance at 340 nm [17]. Matrix-matched blank controls containing identical CA, CS, and buffer without MP were prepared for baseline correction to eliminate spectral interference. All measurements were performed in triplicate. The MP samples included blank control, negative control (MP–CA), and MP–CA–CS ternary systems, all diluted to the target concentration with 0.1 mol/L NaCl.

#### 2.4.2. Quantification of Total Thiols

Total thiol concentration was quantified using the DTNB method [18]. MP samples were prepared at a concentration of 10 mg/mL in 0.1 M NaCl. An aliquot (1 mL) was mixed with 8 mL of urea-Tris-glycine buffer (8 M urea, pH 8.0), vortexed vigorously for 2 min, and then centrifuged at 10,000× *g* for 15 min at 4 °C. A 4.5 mL aliquot of the supernatant was mixed with 0.5 mL of 50 mM DTNB and incubated for 30 min in the dark at ambient temperature; absorbance was then recorded at 412 nm. Matrix blanks without MP but with equivalent CA and CS were used to deduct optical interference. The concentration of thiol groups was determined using a molar absorptivity of 13,600 L·mol^−1^·cm^−1^ and expressed in units of nmol per mg of protein.

#### 2.4.3. Surface Hydrophobicity Measurement

The surface hydrophobicity was assessed using the bromophenol blue (BPB) binding assay [19]. Matrix-matched blanks containing buffer, corresponding concentrations of CA and CS, but no MP were prepared and used for baseline correction to eliminate interference from direct BPB binding by chitosan and spectral absorption by oxidized chlorogenic acid at 595 nm. MP samples were suspended in 0.02 M phosphate buffer (pH 6.0) to achieve a final concentration of 2.5 mg/mL. A mixture of 4 mL protein solution and 0.4 mL of 1 mg/mL BPB was incubated for 8 min at room temperature, followed by centrifugation at 2000× *g* for 12 min. The supernatant was diluted 10-fold with phosphate-buffered saline (PBS) (20 mM, pH 7.0), and absorbance was measured at 595 nm against the matrix-matched blank (buffer + CA + CS without protein). Surface hydrophobicity was quantified as μg BPB bound per mL sample using the formula:

$${BPB}_{bound}\left(\mu g\right)=400\;\mu g\;\times \frac{A_{control}-A_{sample}}{A_{control}}$$

where *A*_control_ = absorbance of the matrix-matched blank containing buffer, CA, and CS without MP; *A*_sample_ = absorbance of the diluted supernatant from MP–BPB incubation mixtures; and 400 μg corresponds to the total BPB dosage added to each reaction system.

#### 2.4.4. Intrinsic Fluorescence Spectral Analysis

Intrinsic fluorescence was measured using a fluorescence spectrophotometer (Cary Eclipse, Agilent Technologies, Santa Clara, CA, USA) at 25 °C [19]. MP samples were prepared at a concentration of 1 mg/mL in phosphate-buffered saline (20 mM, pH 7.0). The fluorescence intensity was acquired at 310–400 nm with excitation at 295 nm, a scan rate of 1200 nm/min, and excitation/emission slits of 5 nm. The maximum emission wavelength (λmax) and relative fluorescence intensity (FI) were recorded for analysis.

#### 2.4.5. Protein Solubility Assay

The solubility of MP was assessed using centrifugation [20]. MP samples were prepared at a fixed total protein concentration of 2.5 mg/mL and centrifuged at 4 °C for 30 min at 5500× *g*. The protein concentration in the supernatant was measured using the Biuret assay. Solubility was calculated as the percentage of protein in the supernatant relative to the total protein.

#### 2.4.6. Circular Dichroism (CD) Spectroscopy

CD spectra were recorded on a JASCO J-815 spectropolarimeter (Jasco Corp., Tokyo, Japan) using a 0.1 cm quartz cuvette at 25 °C. MP samples were diluted to 0.1 mg/mL with 20 mM phosphate buffer (pH 7.0) containing 0.1 M NaCl. Spectra were collected over the far-UV range of 200–260 nm at a scan rate of 50 nm/min, with a bandwidth of 1 nm and response time of 1 s. Three consecutive scans were averaged, and baseline correction was performed using the corresponding buffer solution. The secondary structure compositions (α-helix, β-sheet, β-turn, and random coil) were calculated using the SELCON3 algorithm in the CDPro software (Version 3.0).

### 2.5. Preparation of MP-Based Oil-in-Water Emulsions

Oil-in-water (O/W) emulsions were prepared using the MP samples at 25 °C, following a previously reported method [21]. Briefly, soybean oil was incorporated into the corresponding MP suspensions (adjusted to a fixed total protein concentration of 5 mg/mL with 0.1 mol/L NaCl, CS adjusted to maintain 0.125–1.0 g/g protein) at a volume fraction of 20% (*v*/*v*). The mixtures were homogenized using a digital high-shear homogenizer (HRF-35, Huxi Instrument Co., Ltd., Shanghai, China) via two-stage homogenization: pre-homogenization at 8000 rpm for 30 s to preliminarily disperse oil droplets, followed by high-shear homogenization at 10,000 rpm for 60 s to achieve a finer emulsion structure. Throughout the homogenization process, the sample container was immersed in an ice-water bath to strictly control the temperature below 10 °C, preventing thermal denaturation of MP and ensuring emulsion stability. Freshly prepared emulsions were immediately used for subsequent property determinations, and a portion was stored at 4 °C for stability testing.

### 2.6. Emulsion Properties

#### 2.6.1. Particle Size and Zeta Potential Measurement

The droplet size distribution and zeta potential of the emulsions were measured at 25 °C using a BeNano 90 Zeta nanoparticle size and zeta potential analyzer (Bettersize Instruments Ltd., Dandong, China). Freshly prepared emulsions (containing 5 mg/mL protein) were diluted 1:100 with deionized water prior to analysis. The volume-weighted mean diameter (D_4,3_), surface-area-weighted mean diameter (D_3,2_), and zeta potential were measured sequentially.

#### 2.6.2. Morphological Examination of Emulsions

The microstructure of emulsions was further characterized by fluorescence inverted microscopy (ECLIPSE Ts2R, Nikon Instruments Co., Ltd., Shanghai, China). Nile Blue and Nile Red were used for co-staining. In detail, 30 μL of 0.1% Nile Blue solution and 20 μL of 0.01% Nile Red solution were added to fresh emulsions and incubated for 20 min. A 5 μL aliquot of the mixture was placed on a glass slide, covered with a coverslip and sealed with nail polish. Images were acquired at 200× magnification under unified optical settings.

#### 2.6.3. Emulsion Stability

##### Creaming Index (CI)

Emulsions from each group were transferred into transparent centrifuge tubes and stored at 4 °C for 12 h. After storage, the initial total height of the emulsion (*H*_0_) and the height of the clear subnatant (*H*_1_) were recorded. The creaming index was calculated as:

$$CI\left(\%\right)=\frac{H_{1}}{H_{0}}\times 100$$

##### Emulsifying Activity Index (EAI) and Emulsifying Stability Index (ESI)

For each group, 200 μL of freshly prepared emulsion was carefully pipetted from the bottom of the container and transferred into 20 mL of 0.1% SDS solution, followed by vortexing for 30 s to ensure uniform dispersion. Absorption intensity was measured at a wavelength of 500 nm (denoted as A_0_) using UV-vis spectrophotometry. Matrix-matched blanks containing equivalent CA, CS and SDS solution without emulsion were used to eliminate optical interference. A second measurement was performed after a 10 min incubation at ambient temperature (denoted as A_10_). The EAI and ESI values were determined using the equations below:

$$EAI(m^{2}/g)=\frac{A_{0}\times k\times 2.303\times 2}{C\times \left(1-\varphi \right)\times 10,000}$$

$$ESI(\%)=\frac{A_{10}}{A_{0}}\times 100$$

where C = protein concentration (g/mL), φ = volume fraction of oil (0.2), k indicates dilution factor, l = path length (1 cm), and A_0_ and A_10_ correspond to absorbance at 0 and 10 min separately. All measurements were performed in triplicate.

#### 2.6.4. Rheological Characteristics

Rheological properties were assessed using a Discovery HR-2 rheometer (TA Instruments, New Castle, DE, USA) equipped with a 40 mm parallel-plate geometry and a 1 mm gap, following modified published protocols [16]. All MP samples were standardized to a fixed total protein concentration of 20 mg/mL using a 0.6 M NaCl solution and subsequently incubated at 25 °C for 15 min to ensure thermal equilibrium prior to analysis. To minimize water loss, a thin layer of silicone oil was applied around the periphery of each specimen throughout all experimental procedures.

To identify the linear viscoelastic region (LVR), oscillatory strain sweep tests were performed at 25 °C under a constant angular frequency of 1 Hz, with strain amplitudes ranging from 0.01% to 100%. Dynamic mechanical properties were characterized by measuring the storage modulus (G′) and loss modulus (G″). The LVR was defined as the strain range in which G′ varied by less than 5%. A strain of 0.3%, confirmed within the LVR for both MP emulsions and heat-induced gels, was selected for all subsequent dynamic rheological measurements to avoid structural damage.

##### Apparent Viscosity Determination

The apparent viscosity of the MP samples was determined using a steady-shear rheological test performed at 25 °C, with the shear rate logarithmically increased from 0.1 to 100 s^−1^. The apparent viscosity (η, Pa·s) was recorded continuously to characterize the shear-thinning behavior and dispersion/aggregation state of MP under different CA and CA + CS treatments.

##### Temperature Sweep Test

A temperature sweep was conducted at a strain of 0.3% and a frequency of 1 Hz. The temperature was ramped from 20 to 80 °C at a rate of 2 °C/min. Real-time measurements of the storage modulus (G′) and loss modulus (G″) were used to monitor the thermally induced gelation of MP, capturing key stages including protein unfolding, intermolecular association, and the formation of a three-dimensional gel network.

### 2.7. Gel Properties

#### 2.7.1. Gel Preparation

MP gel preparation was carried out using a classic thermal-induced gelation method, with minor modifications [13]. Cylindrical glass vials were filled with 15 mL of MP suspension that contained a fixed total protein concentration of 40 mg/mL and CS at 0.125, 0.25, 0.5, 1.0 g/g protein. Subsequently, they were subjected to controlled heating in a water bath, where the temperature was ramped from 20 °C to 78 °C at a rate of 2 °C per minute to induce thermal gelation. Following thermal treatment, the gels were allowed to cool to ambient temperature over a 2 h period, refrigerated at 4 °C for approximately 12 h, and then equilibrated at 25 °C for 30 min prior to characterization.

#### 2.7.2. Gel Strength Determination

Gel strength was determined using a Texture Analyzer (model TA-XT, Stable Micro Systems Ltd., Surrey, UK) equipped with a cylindrical P/0.5 probe. The measurement protocol specified pre-test, test, and post-test speeds of 1 mm/s, a trigger force of 5 g, and a total compression depth of 5 mm. Gel strength was defined as the maximum force (N) required to compress the gel. All measurements were performed in triplicate, with three different positions tested per gel.

#### 2.7.3. Water-Holding Capacity

The WHC was measured using centrifugation. An empty centrifuge tube was weighed (*M*_1_), and 5–10 g of gel (from each group) (*M*_0_) was carefully placed into the tube. The sample tube was centrifuged at 4 °C for 10 min at a relative centrifugal force of 3500× *g*. After centrifugation, the upper liquid layer was carefully removed, and the residual moisture on the gel surface was gently absorbed with absorbent filter paper. The tube containing the residual gel was weighed again (designated as *M*_2_), and the WHC was then calculated using the following formula:

$$WHC\left(\%\right)=\frac{M_{2}-M_{1}}{M_{0}}\times 100\%$$

#### 2.7.4. Water Mobility and Distribution

The mobility and distribution of water in MP gels were determined using a LF-NMR apparatus (Niumag Analytical Instrument Co., Shanghai, China) at a magnetic field strength of 0.47 T with a corresponding proton resonance frequency of 20 MHz, according to the method described by Gilbert and Turgeon [22]. A gel sample weighing approximately 2 g was placed into a cylindrical NMR tube with a diameter of 15 mm prior to measurement. The transverse relaxation time (*T*_2_) data were acquired using the Carr–Purcell–Meiboom–Gill (CPMG) pulse sequence, with the following acquisition parameters: waiting time (TW) = 3500 ms, echo time (TE) = 1 ms, number of scans (NS) = 4, and number of echoes (NECH) = 18,000.

### 2.8. Statistical Analysis

All experimental data related to the structural, emulsifying, and gel properties of MP, including water distribution parameters, were analyzed using two-way analysis of variance (ANOVA). The main effects of CA and CS concentrations, as well as their interaction (CS × CA), were evaluated. CA was set at three levels (0, 50, 100 μmol/g protein), and CS at five levels (0, 0.125, 0.25, 0.5, 1.0 g/g protein). Three independent batches of samples were prepared for the entire experiment, and for each batch, three parallel instrumental measurements were performed for every parameter. Results were expressed as mean values ± standard error (SE). Significant differences among all treatment groups were identified by Tukey’s multiple comparison test at a significance level of *p* < 0.05. Statistical analysis was conducted using Statistix version 10.0 (Analytical Software, Tallahassee, FL, USA).

## 3. Results and Discussion

### 3.1. Structural Properties of MP

#### 3.1.1. Turbidity

**Figure 1:**
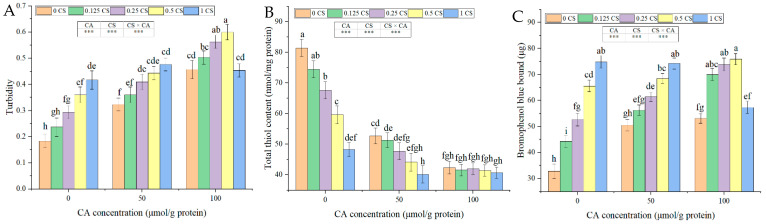
Effects of CS on CA-modified MP: turbidity (**A**), total thiol content (**B**), and surface hydrophobicity (**C**). Error bars indicate the standard error of the mean (SEM). CA, chlorogenic acid; CS, chitosan; CS × CA, Statistical interaction between chitosan and chlorogenic acid. *** *p* < 0.001. Different lowercase letters above bars denote significant differences between groups at *p* < 0.05.

Turbidity serves as an indicator of the aggregation and dispersion stability of MP particles [23]. A significant interaction between CA and CS was detected in relation to MP turbidity (*p* < 0.001). As depicted in Figure 1A, the turbidity of the control group was 0.18. CA led to an increase in turbidity in a dose-dependent manner, reaching 0.32 at 50 μmol/g and 0.46 at 100 μmol/g (*p* < 0.05), which suggests that oxidative damage to MP induced by high-dose CA facilitated intermolecular cross-linking and the formation of insoluble aggregates [8,20]. In the absence of CA, the turbidity gradually increased with the increasing concentration of CS (*p* < 0.05), which can be attributed to the electrostatic attraction between cationic CS and anionic MP [24,25]. In the 50 μmol/g CA group, CS further augmented the turbidity (*p* < 0.05), exacerbating the aggregation of mildly oxidized MP. In the 100 μmol/g CA group, low-to-moderate concentrations of CS (0.125–0.5 g/g protein) increased the turbidity, while a concentration of 1.0 g/g protein significantly reduced it to 0.45 (*p* < 0.05). Low concentrations of CS tended to produce loose, large-particle assemblies, corresponding to elevated light-scattering signals. The decreased turbidity observed at the highest CS concentration implied an altered particle-packing state, which may be associated with tighter intermolecular associations among MP, CA, and CS, and consequently, weakened light-scattering intensity. Unlike most anionic polysaccharides that suppress aggregation via electrostatic repulsion [26,27], CS exhibited a biphasic regulatory effect exclusive to bulk free protein aggregation under severe CA-induced oxidative damage (100 μmol/g CA) in this system. Such biphasic fluctuation only arose from electrostatic cross-linking between low/moderate CS and discrete unfolded MP in aqueous solution, and this rule cannot be generalized to all protein-related properties.

#### 3.1.2. Total Thiol Content

Free thiol groups serve as crucial reactive sites in MP, actively participating in structural unfolding and gel network formation through disulfide cross-linking [28]. A significant interaction between CA and CS was observed in terms of total thiol content (*p* < 0.001). CA-induced oxidative damage to MP led to thiol loss in a dose-dependent manner. CS also reduced thiol content under non-oxidized and mild oxidation conditions, while it exerted little effect on thiol groups of severely oxidized MP. As depicted in Figure 1B, all treatments significantly reduced the thiol content compared to the control group (*p* < 0.05). The control value was 81.44 nmol/mg protein. When CA was used alone, it depleted thiols to 52.69 and 42.34 nmol/mg protein at concentrations of 50 and 100 μmol/g, respectively. In samples without CA, when the concentration of CS was gradually increased, the thiol content decreased to 48.25 nmol/mg protein at 1.0 g/g protein CS. This decrease was attributed to the enhanced electrostatic complexation between CS and MP [16]. At a CA concentration of 50 μmol/g, a CS ratio of 0.125 g/g protein did not significantly change the thiol levels (*p* > 0.05), while higher CS concentrations further reduced the thiol content. In contrast, at a CA concentration of 100 μmol/g, CS did not significantly alter the thiol content (*p* > 0.05), indicating that severe oxidation weakened the regulatory effect of CS. The depletion of detectable free thiols results from multiple synergistic pathways. First, CA undergoes oxidative conversion to electrophilic quinone derivatives, which readily form stable thiol–quinone covalent adducts with cysteine residues on MP and irreversibly consume free thiol groups [9,28]. Second, CA-induced oxidation accelerates intermolecular disulfide cross-linking between MP molecules, converting reactive free thiols into inaccessible disulfide bonds and further reducing measurable thiol signals. Third, positively charged CS interacts electrostatically with negatively charged oxidized MP, covering surface thiol sites and hindering DTNB reagent accessibility, which artificially lowers detected thiol concentrations without permanent chemical consumption of thiols [29]. In severely oxidized MP, massive irreversible protein aggregation creates compact aggregates that internally bury thiol residues; this steric barrier restricts the contact between CS and MP surface groups, thus weakening CS’s shielding effect on thiols [30].

#### 3.1.3. Surface Hydrophobicity

Surface hydrophobicity reflects the tertiary structural unfolding of MP and is closely related to gel performance [31]. A significant interaction between CA and CS was observed in terms of surface hydrophobicity (*p* < 0.001), and both factors significantly promoted the exposure of hydrophobic amino acid residues. As shown in Figure 1C, the control group had a hydrophobicity of 32.87 μg/mL. CA at concentrations of 50 and 100 μmol/g significantly increased the hydrophobicity to 50.57 and 53.13 μg/mL, respectively. There was no significant difference between these two concentrations, indicating a saturation effect. In the absence of CA or under mild oxidation conditions (50 μmol/g CA), CS enhanced the hydrophobicity in a concentration-dependent manner, suggesting progressive unfolding of MP [13]. Under severe oxidation conditions (100 μmol/g CA), CS exerted a biphasic effect on the surface hydrophobicity of MP. The hydrophobicity increased to 75.93 μg/mL at 0.5 g/g protein CS, and then dropped markedly to 57.37 μg/mL at 1.0 g/g protein CS. Low-to-medium CS dosages induced further unfolding of oxidized MP, while 1.0 g/g protein CS likely forms compact MP–CA–CS aggregates via electrostatic attraction, which may mask exposed hydrophobic residues. These results revealed the unique regulatory mechanism of cationic CS in the MP–polyphenol system [8,32].

#### 3.1.4. Intrinsic Fluorescence Spectroscopy

**Figure 2:**
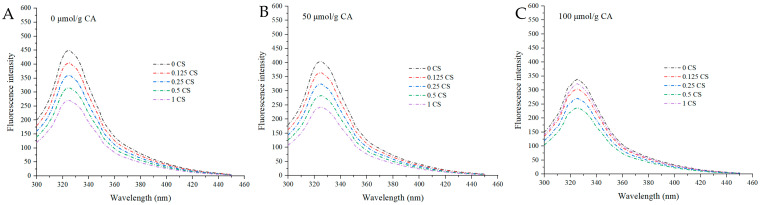
Effects of CS on fluorescence intensity of CA-modified MP. Panels (**A**–**C**) represent samples containing 0, 50, and 100 μmol/g CA, respectively. CA, chlorogenic acid; CS, chitosan.

The intrinsic fluorescence of MP primarily originates from tryptophan residues within the hydrophobic core. Alterations in fluorescence intensity (FI) and the maximum emission wavelength (λ_max_) can sensitively reflect the rearrangement of the tertiary structure [33]. As depicted in Figure 2A–C, both CA and CS significantly modified FI and λ_max_ (*p* < 0.05). When used alone, CA led to a decrease in FI and caused a distinct red-shift in λ_max_ in a dose-dependent manner. This was attributed to protein unfolding and fluorescence quenching by the bound CA. In the absence of CA, an increase in the CS concentration gradually reduced FI and induced a red-shift (*p* < 0.05). This phenomenon likely arises from electrostatic attraction between CS and MP, which disrupts the native compact conformation and shifts the microenvironment of tryptophan residues toward greater solvent exposure [34]. A similar trend was observed in the 50 μmol/g CA group. In the 100 μmol/g CA group, CS exhibited a biphasic regulatory effect. Low-to-moderate CS (0–0.5 g/g protein) further decreased FI and caused a red-shift, whereas the 1.0 g/g protein CS group significantly restored FI and induced a slight blue-shift (*p* < 0.05). The elevated FI and blue-shifted λ_max_ observed at the highest CS concentration indicate a less polar microenvironment surrounding the tryptophan residues, which may result from enhanced intermolecular interactions among MP, CA, and CS. These results were consistent with the surface hydrophobicity, which demonstrated that the regulatory effect of CS on the tertiary structure of MP varied with the CS dosage and the oxidation degree.

#### 3.1.5. Protein Solubility

**Figure 3:**
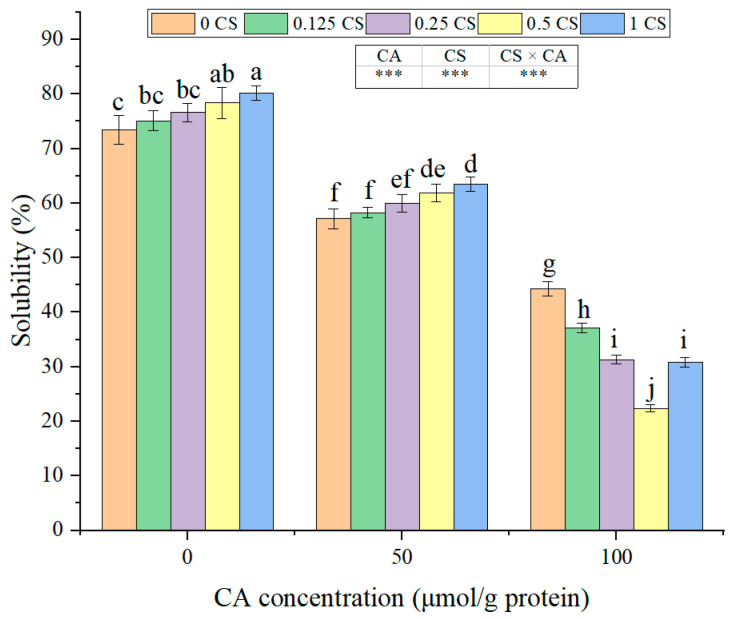
Effects of CS on protein solubility of CA-modified MP. Under each CA concentration (0, 50, and 100 μmol/g protein). The error bars indicate the standard error of the means (SEM). CA, chlorogenic acid; CS, chitosan; CS × CA, Statistical interaction between chitosan and chlorogenic acid. *** *p* < 0.001. Different lowercase letters above bars denote significant differences between groups at *p* < 0.05.

Protein solubility reflects the structural stability and dispersibility of MP [26]. A two-way ANOVA indicated that CA and CS exerted extremely significant main effects on solubility (*p* < 0.001), and a highly significant CA × CS interactive effect was also detected (*p* < 0.001). This finding demonstrates that the regulatory influence of CS on solubility varied markedly under different CA oxidation levels. As shown in Figure 3, CA significantly reduced solubility in a dose-dependent manner (*p* < 0.001). CA-induced oxidative damage to MP triggered protein unfolding and the exposure of hydrophobic domains, leading to excessive aggregation and decreased hydration, thus reducing solubility [8,35]. CS exerted distinct effects on solubility across different degrees of oxidation. Under non-oxidative (0 μmol/g protein CA) and mild oxidative (50 μmol/g protein CA) conditions, the solubility increased gradually as the CS dosage increased (*p* < 0.001). Under non-oxidative conditions, CS enhanced hydrophilicity and electrostatic repulsion, thereby hindering protein aggregation [16]. For moderately oxidized MP, CS adsorbed onto protein surfaces, disperses aggregates, and re-establishes protein–water interactions to increase solubility [36]. For severely oxidized MP (100 μmol/g protein CA), low and medium dosages of CS (0.125–0.5 g/g protein) led to a further reduction in solubility when compared with the corresponding samples treated with CA alone. The minimum solubility value was recorded at a CS dosage of 0.5 g/g protein. Only the highest CS dosage (1.0 g/g protein) led to a partial increase in solubility. However, its value still remained significantly lower than that of the CA-only group at 100 μmol/g CA. These trends indicate that the increasing concentrations of CS gradually enhance solubility under both non-oxidative and mild oxidative conditions. Conversely, low-to-medium levels of CS exacerbated the loss of solubility under severe CA-induced oxidative stress, and even the maximum dosage of CS was unable to fully restore the solubility of severely oxidized MP.

Consistent biphasic trends were observed simultaneously for turbidity, surface hydrophobicity, tertiary structure, and solubility exclusively under severe CA-induced oxidative damage (100 μmol/g protein CA). At low-to-moderate CS dosages, cationic CS may act as electrostatic bridges to cross-link multiple unfolded, oxidatively damaged MP molecules, which likely form bulky loose aggregates; such structural changes could raise turbidity and surface hydrophobicity, disrupt the microenvironment of tryptophan residues, and further lower protein solubility [16,36]. At the maximum CS dosage (1.0 g/g protein), abundant positively charged CS may fully cover the surfaces of protein aggregates, shielding exposed hydrophobic residues and mitigating excessive particle aggregation. This behavior could partially restore solubility and intrinsic fluorescence intensity while reducing turbidity, which aligns with previous reports regarding polysaccharide encapsulation of oxidized protein aggregates [13,26]. Nevertheless, CS supplementation appears unable to fully reverse irreversible structural damage to MP triggered by CA-derived quinone oxidation. In contrast, under non-oxidative (0 μmol/g protein CA) and mild CA-induced oxidative damage (50 μmol/g protein CA), the four aggregation-related indicators shifted monotonically with increasing CS concentration without biphasic fluctuations. This distinction may suggest that CS could display two divergent regulatory tendencies depending on oxidation severity and the type of measured aggregation indicators: biphasic responses only emerge for MP aggregation-related indicators under severe oxidation, whereas CS may gradually mitigate protein structural disorder and aggregation under low and moderate oxidation levels, which is presumably driven by sustained electrostatic interactions between CS and MP [16,36].

#### 3.1.6. Secondary Structure Analysis

**Figure 4:**
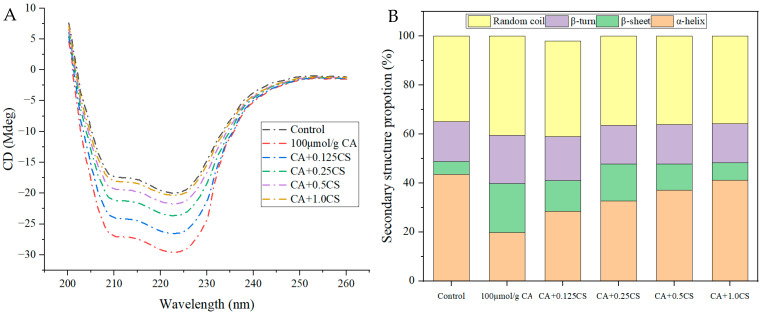
Circular dichroism spectra (**A**) and secondary structure composition (**B**) of porcine MP under different treatments. Control: untreated MP; 100 μmol/g CA: MP treated with 100 μmol/g chlorogenic acid; CA + 0.125CS, CA + 0.25CS, CA + 0.5CS, CA + 1.0CS: MP treated with 100 μmol/g CA and 0.125, 0.25, 0.5, 1.0 g CS/g protein, respectively.

Given that 100 μmol/g CA resulted in the most severe structural damage, the far-UV CD spectra of the control group, the 100 μmol/g CA group, and the CS-supplemented groups were compared (Figure 4). All samples exhibited typical double negative peaks at 208 nm and 222 nm, which corresponded to the α-helical structure of MP. CA-induced oxidative damage to MP significantly reduced the peak intensity, suggesting obvious α-helix loss and protein unfolding. CS gradually restored the peak intensity in a dose-dependent manner. The low-dose CS (0.125 g/g protein) only marginally improved the spectrum, whereas higher concentrations clearly mitigated the secondary structure damage. The 1.0 g/g protein CS group presented a spectral profile approaching that of the control. Quantitative analysis revealed that the α-helix content was 43.5% in the control, but it sharply declined to 19.8% after CA-induced oxidative treatment (*p* < 0.05), while the β-sheet and random coil contents increased significantly. As the CS concentration increased, the α-helix content gradually recovered to 28.4%, 32.6%, 37.0%, and 41.2% at 0.125, 0.25, 0.5, and 1.0 g/g protein, respectively, and the β-sheet and random coil contents decreased correspondingly. These spectral observations indicate that CA-induced oxidative damage disrupted the ordered α-helical conformation of MP, and CS treatment partially recovered secondary structural features in a concentration-dependent trend by inhibiting unfolding and preserving the α-helix, which was consistent with the changes in emulsifying properties. Notably, different from the aggregation behavior of MP, CS restored the secondary structure in a continuous dose-dependent manner without adverse effects at low concentrations.

### 3.2. Properties of MP-Based Oil-in-Water Emulsions

#### 3.2.1. Particle Size and Zeta Potential

Droplet size and zeta potential serve as crucial indicators for the evaluation of emulsion stability and interfacial electrostatic interactions [37]. As presented in Table 1, CA, CS, and their interaction had a significant impact on emulsion properties. The D_4,3_ value was significantly affected by CA, CS, and their interaction (*p* < 0.001). The D_3,2_ value was significantly influenced by CA and the interaction (*p* < 0.001), whereas CS had a marginal effect (*p* = 0.054). Regarding zeta potential, CA showed a significant effect (*p* = 0.001), and CS and the interaction were extremely significant (*p* < 0.001). The regulatory effect of CS on emulsion stability depended on both CA concentration and CS dosage: low-to-moderate CS promoted droplet aggregation under mild oxidation, while high-dose CS inhibited excessive aggregation. The control group displayed a D_4,3_ of 1.79 μm and a D_3,2_ of 0.72 μm. CA significantly increased the droplet size (*p* < 0.05), with the D_4,3_ reaching 3.45 and 5.27 μm at 50 and 100 μmol/g CA, respectively. An increase in particle size is a typical manifestation of droplet flocculation and aggregation in emulsions [38]. CA-induced oxidative damage to MP exposed the hydrophobic regions of protein molecules and promoted the formation of large aggregates, thus weakening the interfacial adsorption ability and emulsifying performance [38]. In CA-free emulsions, CS increased the droplet size (*p* < 0.05) because of the electrostatic complexation between cationic CS and anionic MP. In the 50 μmol/g CA group, low-to-medium CS further enlarged the droplets, while 1.0 g/g protein CS significantly reduced the D_4,3_. In the 100 μmol/g CA group, the droplet size reached its peak at 0.25 g/g protein CS and then decreased, with 1.0 g/g protein CS resulting in the smallest droplets (*p* < 0.05).

Zeta potential serves as a crucial index that reflects the surface charge characteristics and electrostatic repulsion within emulsion systems. A larger absolute zeta potential indicates a stronger electrostatic repulsion between droplets, which is beneficial to dispersion stability [21]. The control emulsion exhibited a zeta potential of −28.20 mV, indicating a strong negative charge on the MP droplets. CA significantly increased the absolute value (*p* = 0.001), while CS neutralized the surface charge and decreased the absolute potential in a concentration-dependent manner (*p* < 0.001). For samples containing 100 μmol/g CA and CS at 1.0 g/g protein, the zeta potential was approximately −1.67 mV. Although the electrostatic repulsion was weakened, the strong steric hindrance provided by the adsorbed CS layers at the oil–water interface effectively prevented droplet aggregation and maintained excellent emulsion stability [36]. Similar findings have confirmed that cationic CS interacts with anionic MP via electrostatic attraction, regulates the surface charge properties, and enhances the stability of protein-based dispersions [21,36].

#### 3.2.2. Microstructure

**Figure 5:**
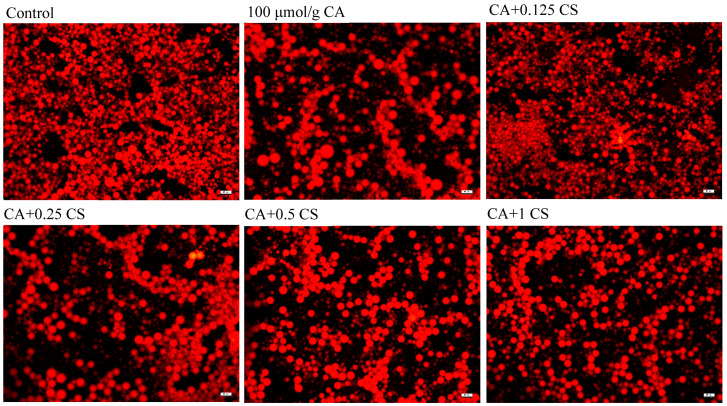
Microstructure of MP emulsions. Control, 100 μmol/g CA, CA + 0.125 CS, CA + 0.25 CS, CA + 0.5 CS, and CA + 1.0 CS correspond to the blank control, 100 μmol/g CA treatment, and CA treatments supplemented with 0.125, 0.25, 0.5, and 1.0 g/g protein CS, respectively.

Microstructure can visually depict the dispersion state of emulsion droplets and validate the results of particle size analysis [39]. Fluorescence microscopy (Figure 5) visually verified these changes. The control sample displayed uniform and fine droplets, while 100 μmol/g CA led to severe aggregation and the formation of irregular flocs. A low concentration of CS (0.125 g/g protein) had little effect on improving the microstructure. As the CS concentration increased, the dispersion of the droplets gradually improved. The group with CS at 1.0 g/g protein exhibited the most uniform structure, which was similar to that of the control. High CS dosages tended to reduce droplet aggregation in emulsions, which implies that electrostatic interactions between MP, CA and CS may generate compact interfacial aggregates and partially strengthen the interfacial layer [21]. In contrast, low concentrations of CS were unable to fully cover the surface of aggregates and thus could not effectively mitigate aggregation. These results revealed that the restorative capacity of CS on emulsion microstructure gradually strengthened with increasing dosage, and an obvious improvement was observed at high CS concentrations.

#### 3.2.3. Creaming Index

**Figure 6:**
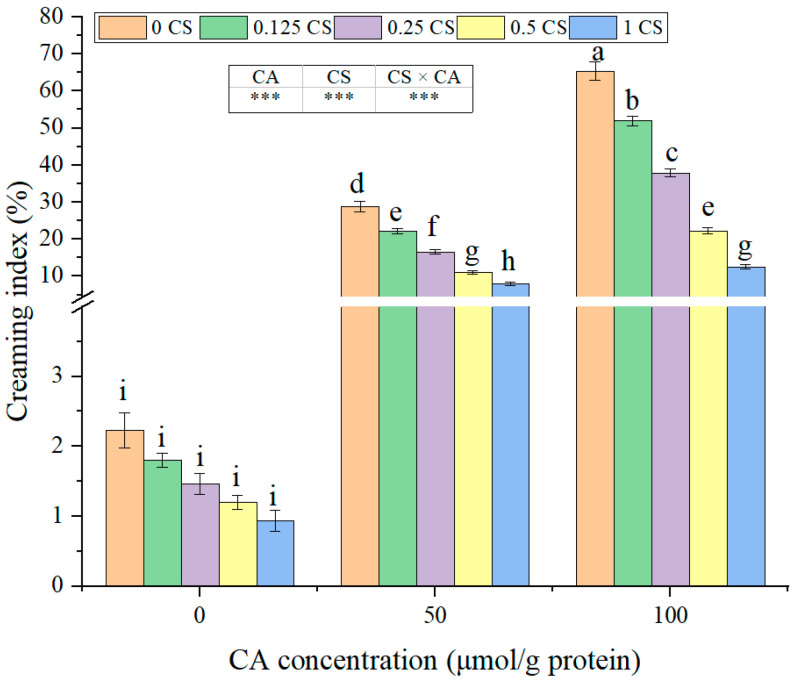
Effects of CS on creaming index of CA-modified MP. respectively. Error bars indicate the standard error of the mean (SEM). CA, chlorogenic acid; CS, chitosan; CS × CA, Statistical interaction between chitosan and chlorogenic acid. *** *p* < 0.001. Different lowercase letters above bars denote significant differences between groups at *p* < 0.05.

The creaming index (CI) was employed to assess the phase separation and storage stability of the emulsion after 12 h of storage at 4 °C. A significant interaction between CA and CS was detected for the creaming index (*p* < 0.001). CA notably impaired the stability of the emulsion, whereas CS effectively mitigated the phase separation. As depicted in Figure 6, the control exhibited excellent stability, with a creaming index of 2.0%. CA significantly elevated the creaming index in a dose-dependent manner (*p* < 0.05), reaching 28.8% at 50 μmol/g and 65.4% at 100 μmol/g. CA-induced oxidative damage to MP triggered protein aggregation, weakened the emulsifying capacity and expedited the flocculation and stratification of droplets. In emulsions without CA, CS gradually decreased the creaming index as its concentration increased (*p* < 0.05). In the 50 μmol/g CA group, CS reduced the creaming index from 28.8% to 7.9% at 1.0 g/g protein CS (*p* < 0.05). In the 100 μmol/g CA group, CS more significantly reduced the creaming index from 65.4% to 12.5%, and almost no stratification was observed at 1.0 g/g protein CS. In line with previous reports [36,40], CS improved emulsion stability by suppressing excessive droplet aggregation. This stabilizing effect may arise from strengthened intermolecular interactions among MP, CA, and CS, which inhibit droplet flocculation and phase separation.

#### 3.2.4. EAI and ESI

**Figure 7:**
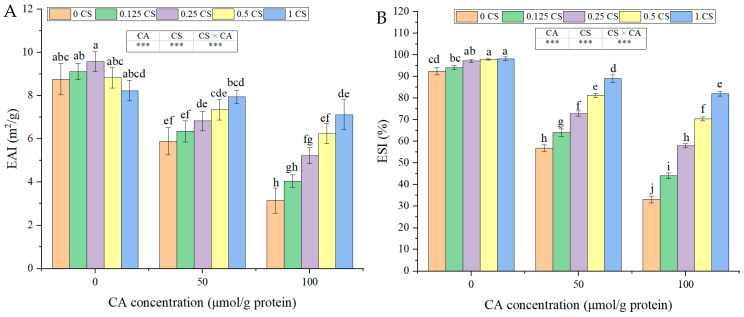
Effect of CS on EAI (**A**) and ESI (**B**) of CA-modified MP. Error bars indicate the standard error of the means (SEM). CA, chlorogenic acid; CS, chitosan; CS × CA, Statistical interaction between chitosan and chlorogenic acid. *** *p* < 0.001. Different lowercase letters above bars denote significant differences between groups at *p* < 0.05.

EAI reflects the efficiency of protein adsorption during the emulsification process, while ESI indicates the emulsion’s resistance to flocculation and coalescence [41]. A significant interaction between CA and CS was observed for both indices (*p* < 0.001). CA severely impaired the emulsifying properties, whereas CS effectively alleviated these detrimental effects. As depicted in Figure 7A,B, the control sample exhibited an EAI of 8.76 m^2^/g and an ESI of 92.35%. Both indices decreased in a dose-dependent manner with the addition of CA (*p* < 0.001). Specifically, at a CA concentration of 50 μmol/g, the EAI dropped to 5.89 m^2^/g and the ESI to 57.23%, and further decreased to 3.15 m^2^/g and 33.52%, respectively, at a CA concentration of 100 μmol/g. This phenomenon was attributed to the CA-induced oxidative damage to MP, which causes protein unfolding and aggregation and impairs interfacial adsorption and structural stability [20,38]. In CA-free emulsions, CS had a significant influence on EAI and ESI (*p* < 0.001). The EAI first increased slightly and then decreased, while the ESI rose continuously as the CS concentration increased. In the 50 μmol/g CA group, CS enhanced the EAI and ESI in a dose-dependent manner (*p* < 0.05). When the CS concentration was 1.0 g/g protein, the EAI and ESI reached 7.95 m^2^/g and 89.67%, respectively. In the 100 μmol/g CA group, CS enhanced the emulsification more remarkably. At a CS concentration of 1.0 g/g protein, the EAI and ESI increased to 7.12 m^2^/g and 82.37%, respectively (*p* < 0.05). High-concentration CS facilitated the formation of compact interfacial complexes, thereby strengthening the interfacial adsorption and emulsion stability. Unlike the biphasic changes in protein aggregation, CS improved emulsifying performance in a steady dose-dependent manner.

### 3.3. Rheological Characteristics of MP

#### 3.3.1. Apparent Viscosity

**Figure 8:**
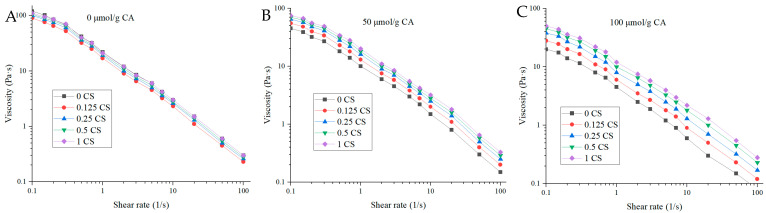
Effects of CS on apparent viscosity of CA-modified MP. Panels (**A**–**C**) represent samples containing 0, 50, and 100 μmol/g CA, respectively. CA, chlorogenic acid; CS, chitosan.

Apparent viscosity reflects the flow resistance of MP systems and plays a crucial role in processing optimization and quality evaluation [42]. A significant interaction between CA and CS was observed in terms of apparent viscosity (*p* < 0.001). Specifically, CA significantly reduced the viscosity, whereas CS effectively restored it. These effects were more pronounced at low shear rates (0.1–10 s^−1^) and, although weakened, still remained significant at high shear rates (50–100 s^−1^). All samples demonstrated typical shear-thinning behavior (Figure 8A–C), which was attributed to the shear-induced disruption of weak intermolecular networks and molecular alignment [20,43]. CA decreased the apparent viscosity in a dose-dependent manner (*p* < 0.05). CA-induced oxidative damage to MP causes oxidative unfolding and disordered aggregation, disrupting the continuous protein network and thereby reducing the system’s flow resistance. At the same CA level, the viscosity increased with the CS concentration (*p* < 0.05), and a CS concentration of 1.0 g/g protein resulted in the highest viscosity. The electrostatic complexation between cationic CS and anionic MP led to a denser and more stable network, as well as higher system rigidity. CS exhibited a stronger viscosity-recovering effect in the 100 μmol/g CA group, which may be attributed to enhanced cross-association among MP, CA, and CS, thereby mitigating the network damage induced by severe CA modification. These results were consistent with the emulsion stability and structural changes, further confirming that CS effectively restored the impaired functional properties of MP suffering from CA-induced oxidative damage.

#### 3.3.2. Temperature Sweep

**Figure 9:**
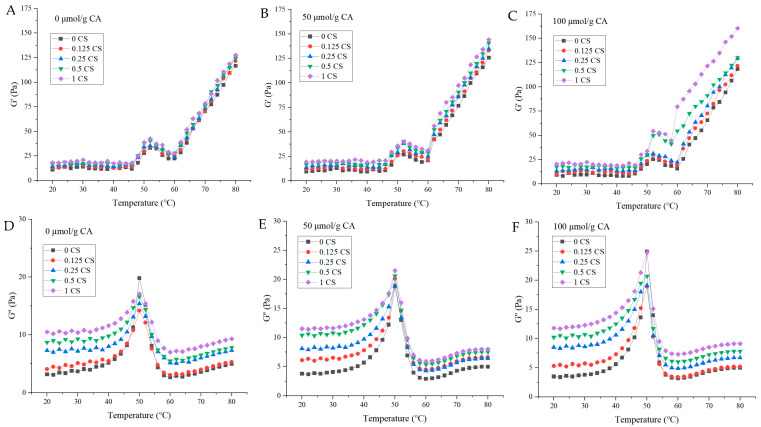
Effects of CS on storage modulus (G′, (**A**–**C**)) and loss modulus (G″, (**D**–**F**)) of CA-modified MP under 0, 50, and 100 μmol/g CA, respectively. CA, chlorogenic acid; CS, chitosan.

The thermal gelation of MP involves an orderly aggregation of proteins to form a continuous three-dimensional network [44]. The storage modulus (G′) and loss modulus (G″) were measured to characterize the thermally induced gelation behavior (Figure 9A–C for G′, Figure 9D–F for G″). In the control group, G′ remained stable when the temperature was below 45 °C, whereas G″ increased gradually. This phenomenon suggests weak intermolecular interactions and indicates that a stable network has not yet formed. A distinct G″ peak emerged at approximately 52 °C, which may correspond to the denaturation of myosin and the initial cross-linking of aggregates. In comparison with the control, 50 μmol/g of CA led to a decrease in the G′ peak and a slight increase in the G″ denaturation peak. The addition of 100 μmol/g of CA further reduced G′ and enhanced G″, which implies that CA may weaken the formation of elastic gels in a dose-dependent manner and potentially promote protein unfolding and viscous dissipation. This phenomenon was consistent with previous reports indicating that the oxidation induced by polyphenols disrupts the regular assembly of MPs during heating, thereby deteriorating the viscoelastic properties of gels [45]. The addition of CS to the 100 μmol/g CA group significantly increased G′ and suppressed the excessive rise in G″, which appears to mitigate the loss of gel elasticity caused by CA-induced oxidative damage. From 60 to 80 °C, the G′ of all samples increased sharply, while G″ decreased and remained at a low level, which may point to complete protein unfolding and the gradual construction of a stable elastic network [46]. At each CA level, the CS-treated groups exhibited higher G′ and more appropriate G″ values, suggesting that CS likely reinforces the elastic network and improves the viscoelastic balance of MP gels. Similar results have been reported in related studies [16,34]. Those studies demonstrated that CS interacts with MP via electrostatic forces to consolidate the gel network structure and optimize the gel’s viscoelastic balance during heating. The improved viscoelasticity observed in the present work may partly stem from similar electrostatic interactions between CS and oxidized MP.

### 3.4. Gel Properties of MP

#### 3.4.1. Gel Strength

**Figure 10:**
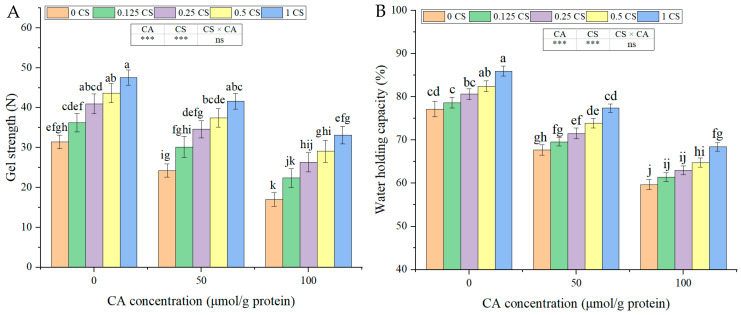
Gel strength (**A**) and water-holding capacity (**B**) of the MP gels with different levels of CS and CA. CA, chlorogenic acid; CS, chitosan; CS × CA, Statistical interaction between chitosan and chlorogenic acid. ns, significant; *** *p* < 0.001. Different lowercase letters above bars denote significant differences between groups at *p* < 0.05.

Gel strength reflects the mechanical properties of MP gels and is closely associated with the compactness and integrity of the gel network [47]. Two-way ANOVA indicated that both CA and CS had extremely significant main effects on gel strength (*p* < 0.001), whereas their interaction was not significant (*p* > 0.05). This finding indicates that the reinforcing potency of CS did not show a statistically significant difference under different CA oxidation levels. As depicted in Figure 10A, CA gradually reduced gel strength in a dose-dependent manner (*p* < 0.001). CA-induced oxidative damage to MP triggers excessive protein unfolding and disordered aggregation, thereby disrupting ordered intermolecular cross-linking and hindering the formation of a compact and continuous gel network [38]. This conclusion is in good accordance with previous findings that polyphenol-mediated oxidation disrupts the regular cross-linking of MPs and weakens the mechanical strength of the final gels [28,48]. In contrast, CS significantly enhanced gel strength in a concentration-dependent manner at all CA levels (*p* < 0.001). In the CA-free group, CS may facilitate the formation of a dense network through electrostatic interaction with MP. Numerous studies have confirmed that cationic chitosan can combine with anionic MPs to construct more rigid network structures and improve the mechanical properties of protein gels [16,34]. In the 50 and 100 μmol/g CA groups, the gel strength increased steadily with the addition of CS. The sample supplemented with CS at 1.0 g/g protein achieved the most significant improvement compared with the CA-alone treatments (*p* < 0.001). Similarly, CS improved gel properties in a dose-dependent manner across all oxidation levels, with no biphasic trend observed.

#### 3.4.2. WHC

WHC is a core indicator of gel quality, which directly determines the sensory and textural properties of meat products [49]. It mainly depends on the compactness and integrity of the MP gel network. Two-way ANOVA demonstrated that both CA and CS had extremely significant effects on gel WHC (*p* < 0.001), while their interaction was not significant (*p* > 0.05), suggesting that CA and CS exert separate additive effects rather than interactive regulation. As depicted in Figure 10B, an increase in CA concentration significantly reduced gel WHC (*p* < 0.001). CA-induced oxidative damage to MP disrupted protein cross-linking and led to the formation of a loose, discontinuous gel network with large pores, thereby weakening the water-trapping capacity and exacerbating water loss during heating [35,50]. CS significantly enhanced WHC in a concentration-dependent manner under all oxidative conditions (*p* < 0.001). In the absence of CA, CS promoted the formation of a compact gel network through electrostatic interaction with MP, thus improving water retention [16]. In the 50 and 100 μmol/g CA groups, increasing CS concentration gradually repaired the damaged gel microstructure: CS filled cracks and pores, promoted ordered molecular cross-linking, reduced water mobility, and ultimately enhanced WHC. The group treated with CS at 1.0 g/g protein exhibited the optimal restorative capacity to alleviate water loss caused by CA-induced oxidative damage, with values significantly higher than those of the corresponding CA-only groups (*p* < 0.001).

#### 3.4.3. Low-Field NMR Relaxation Behavior

**Figure 11:**
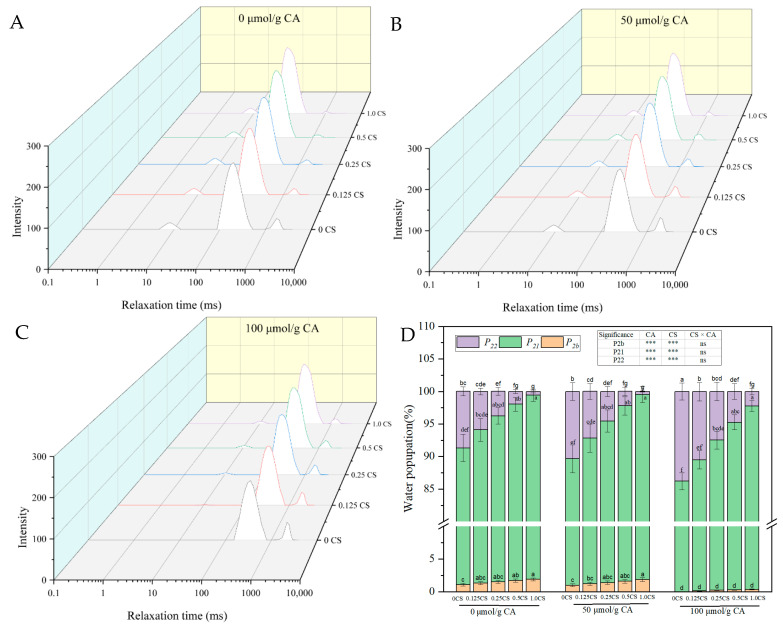
Effects of CS on relaxation time distribution curves (**A**–**C**) and corresponding peak area percentages (**D**) of CA-modified MP. Error bars indicate the standard error of the mean (SEM). CA, chlorogenic acid; CS, chitosan; CS × CA, Statistical interaction between CS and CA. ns, significant; *** *p* < 0.001. Different lowercase letters above bars denote significant differences between groups at *p* < 0.05.

Low-field NMR was employed to characterize the water state and mobility within MP gels. The *T*_2_ relaxation time distribution illustrates water compartmentalization (bound water *T*_2*b*_, immobilized water *T*_21_, free water *T*_22_) and is closely related to gel network integrity and WHC [22]. As depicted in Figure 11A–C, three water populations were identified across all samples. CA extended the *T*_2_ relaxation times in a dose-dependent manner and shifted the *T*_21_ and *T*_22_ peaks to the right, whereas the *T*_2*b*_ peak was significantly attenuated. CA-induced oxidative damage to MP destroyed the protein’s spatial structure, loosened the gel network, weakened protein–water interactions, and increased water mobility. In comparison with the control group, 100 μmol/g CA significantly increased *T*_21_ (351.1 ms) and *T*_22_ (2203.5 ms), and 50 μmol/g CA also elevated both relaxation times (*p* < 0.05). CS effectively reversed these alterations, shortened the *T*_2_ values, and restored the *T*_2*b*_ peak. As the concentration of CS increased, *T*_21_ and *T*_22_ gradually decreased, reaching 174.8/1260.4 ms (100 μmol/g CA) and 114.9/1024.6 ms (50 μmol/g CA) at 1.0 g/g protein CS.

CS increased the proportion of immobilized water (*P*_21_) and decreased that of free water (*P*_22_) in a dose-dependent manner under all treatments (Figure 11D). Two-way ANOVA indicated significant main effects of CA and CS (*p* < 0.001), yet there was no significant interaction (*p* > 0.05). At a CS concentration of 1.0 g/g protein, *P*_21_ increased to 97.40% (at 100 μmol/g CA) and 97.69% (at 50 μmol/g CA), while *P*_22_ decreased sharply (*p* < 0.05). CS trapped free water within the gel network and transformed it into immobilized water, thus enhancing the WHC. A similar effect was also observed in CA-free samples. These results are consistent with previous reports stating that polysaccharides enhance gel compactness and restrict water migration [20,51].

## 4. Conclusions

This study investigated the regulatory roles of chitosan (CS) on myofibrillar protein (MP) subjected to oxidative damage induced by chlorogenic acid (CA) at both macroscopic and interfacial scales, and revealed two different response patterns of CS via multiple spectroscopic and emulsification measurements. The electrostatic binding affinity between cationic CS and MP varies with the extent of CA-triggered oxidative damage, which may produce two differentiated regulatory responses of CS that differ across oxidative degrees and detection indicators. Four aggregation-associated indices, namely turbidity, surface hydrophobicity, tertiary structure, and solubility, only displayed biphasic variations under severe CA-triggered oxidative damage (100 μmol/g protein). Under this oxidative condition, low and moderate CS concentrations may cross-link unfolded MP to form large, loose aggregates, whereas the maximum CS dosage possibly encapsulates the surfaces of aggregates and alleviates excessive protein aggregation. In contrast, these four indices rose progressively with elevated CS levels without biphasic shifts in non-oxidized and mildly oxidized groups (50 μmol/g protein), where CS suppressed oxidative aggregation of MP and preserved well-ordered secondary conformations. Conformational, emulsifying, rheological and gel-related properties generally improved gradually as CS concentration increased, regardless of oxidative damage intensity. Electrostatic complexation between CS and MP could potentially maintain ordered protein conformations, intact oil–water interfacial membranes and compact three-dimensional gel networks. Two-way ANOVA indicated significant interactive effects between CA and CS on protein conformation, turbidity, surface hydrophobicity, solubility, emulsification and rheology (*p* < 0.001), suggesting that the regulatory performance of CS is closely associated with the severity of CA-triggered oxidative damage. No obvious CA–CS interaction was observed for gel strength, water-holding capacity and water distribution (*p* > 0.05), illustrating that CS could stably improve gel properties under all tested oxidative treatments. Among all tested CS gradients, the dosage of 1.0 g/g protein produced the most evident restorative capacity to counteract CA-induced structural and functional deterioration of MP. It should be noted that in this study, three MP batches sourced from different pig individuals were analyzed. However, a wider range of animal individuals, muscle cuts, and meat materials were not considered. These unexamined variations may restrict the practical applicability of the results. The present findings offer supplementary theoretical references for modulating meat MP with CA-triggered oxidative damage using cationic polysaccharides, and provide technical references for the joint application of CA and CS in meat processing.

## Figures and Tables

**Table 1:** Particle size (D_4,3_, D_3,2_) and zeta potential of myofibrillar protein emulsions as affected by chlorogenic acid and chitosan.

	CA (μmol/g Protein)	CS (g/g Protein)					Significance		
		0	0.125	0.25	0.5	1	CA	CS	CS × CA
D_4,3_ (μm)	0	1.79 ^k^	2.14 ^jk^	2.47 ^ij^	2.77 ^hi^	3.13 ^gh^	<0.001	<0.001	<0.001
	50	3.45 ^fg^	3.76 ^ef^	4.14 ^de^	4.35 ^d^	3.69 ^f^			
	100	5.27 ^c^	5.77 ^ab^	6.15 ^a^	5.47 ^bc^	4.19 ^d^			
D_3,2_ (μm)	0	0.72 ^g^	0.75 ^fg^	0.77 ^fg^	0.80 ^defg^	0.83 ^cdefg^	<0.001	0.054	<0.001
	50	0.78 ^efg^	0.81 ^defg^	0.84 ^cdefg^	0.87 ^bcdef^	0.90 ^abcde^			
	100	0.94 ^abc^	0.97 ^ab^	1.00 ^a^	0.91 ^abcd^	0.86 ^bcdef^			
Zeta Potential (mV)	0	−28.20 ^ij^	−22.53 ^fg^	−18.76 ^de^	−12.45 ^c^	−7.82 ^b^	0.001	<0.001	<0.001
	50	−30.15 ^ij^	−24.38 ^gh^	−19.87 ^ef^	−13.69 ^c^	−8.93 ^b^			
	100	−31.52 ^j^	−26.74 ^hi^	−21.45 ^efg^	−15.82 ^cd^	−1.67 ^a^			

Note: Means with different letters indicate statistically significant differences among all groups (*p* < 0.05). D_4,3_ = Volume-weighted mean diameter; D_3,2_ = Surface-weighted mean diameter. CA, CS, and CS × CA represent *p*-values for CA treatment, CS treatment, and their interaction, respectively. CA: chlorogenic acid; CS: chitosan.

## Data Availability

The original contributions presented in this study are included in the article. Further inquiries can be directed to the corresponding author.

## References

[1] Xu Q., Yu Z., Zeng W. (2021). Structural and functional modifications of myofibrillar protein by natural phenolic compounds and their application in pork meatball. Food Res. Int..

[2] Bao Y., Ertbjerg P. (2019). Effects of protein oxidation on the texture and water-holding of meat: A review. Crit. Rev. Food Sci. Nutr..

[3] Cao Q., Huang Y., Zhu Q.-F., Song M., Xiong S., Manyande A., Du H. (2020). The mechanism of chlorogenic acid inhibits lipid oxidation: An investigation using multi-spectroscopic methods and molecular docking. Food Chem..

[4] Yu L., Zhang X., Guo C., Li M., Zeng M. (2024). Inhibitory effect of chlorogenic acid and vanillic acid on fluorescent advanced glycation end products formation in low-temperature-processed pork meat. Food Biosci..

[5] Galić L., Lončarić Z., Lisjak M. (2025). A Review of Phenolic Compounds: From Biosynthesis and Ecological Roles to Human Health and Nutrition. Phyton-Int. J. Exp. Bot..

[6] Chen Y., Jiang Q., Lu Y., Zeng Q., Liu P., Chen J., Lin H., Tang J., Zhao J. (2026). Effect of protein–polyphenol interactions on the structural and digestive properties of pork myofibrillar proteins. J. Food Eng..

[7] Guo X., Qiu H., Deng X., Mao X., Guo X., Xu C., Zhang J. (2019). Effect of Chlorogenic Acid on the Physicochemical and Functional Properties of Coregonus Peled Myofibrillar Protein through Hydroxyl Radical Oxidation. Molecules.

[8] Jia N., Lin S., Zhang F., Zheng D., Liu D. (2022). Improved effect of flaxseed gum on the weakened gelling properties of myofibrillar protein induced by catechin. Food Chem..

[9] Cao Y., Xiong Y.L. (2015). Chlorogenic acid-mediated gel formation of oxidatively stressed myofibrillar protein. Food Chem..

[10] Lv Y., Chen L., Wu H., Xu X., Zhou G., Zhu B., Feng X. (2019). (-)-Epigallocatechin-3-gallate-mediated formation of myofibrillar protein emulsion gels under malondialdehyde-induced oxidative stress. Food Chem..

[11] Wang W., Xue C., Mao X. (2020). Chitosan: Structural modification, biological activity and application. Int. J. Biol. Macromol..

[12] Ravi Kumar M.N.V. (2000). A review of chitin and chitosan applications. React. Funct. Polym..

[13] Zhang H., Li X., Zhang Z., Jiang A., Bai Q. (2025). Effect of chitosan on thermal gelling properties of pork myofibrillar protein and its mechanism. J. Sci. Food Agric..

[14] Gao C., Xing Z., Chen Y., Meng L., Tang X. (2025). Improve the chitosan particle-stabilized oil-water interface by dual reinforcement and its effect on the structure and properties of emulsion. Food Chem..

[15] Anraku M., Gebicki J.M., Iohara D., Tomida H., Uekama K., Maruyama T., Hirayama F., Otagiri M. (2018). Antioxidant activities of chitosans and its derivatives in in vitro and in vivo studies. Carbohydr. Polym..

[16] Huang M., Xu Y., Xu L., Bai Y., Xu X. (2022). Interactions of water-soluble myofibrillar protein with chitosan: Phase behavior, microstructure and rheological properties. Innov. Food Sci. Emerg. Technol..

[17] Zhang Y., Chen L., Lv Y., Wang S., Suo Z., Cheng X., Xu X., Zhou G., Li Z., Feng X. (2018). Inhibition of interaction between epigallocatechin-3-gallate and myofibrillar protein by cyclodextrin derivatives improves gel quality under oxidative stress. Food Res. Int..

[18] Lu Y., Zhuang Y., Jiang Y., Wang J., Dong L., Zhang Y., Wang S. (2025). Impact of lipid oxidation products on the digestibility and structural integrity of Myofibrillar proteins during thermal processing. Food Chem..

[19] Chelh I., Gatellier P., Santé-Lhoutellier V. (2006). Technical note: A simplified procedure for myofibril hydrophobicity determination. Meat Sci..

[20] Chen J., Wang S., Ji Z., Yi X., Guo J., Jin G., Wu Z. (2025). Inhibition mechanisms of xanthan gum on high-dose gallic acid-induced functional deterioration of myofibrillar protein: Focusing on gelling and emulsification behaviors. Carbohydr. Polym..

[21] Zhao Y., Zhao X., Xu X. (2025). Investigating the influence of myofibrillar protein and chitosan interfacial distribution on the macroscopic characteristics of emulsions. Food Chem..

[22] Gilbert A., Turgeon S.L. (2026). Unraveling microstructure and water behavior in diverse food matrices using low-frequency NMR (LF-NMR) on proton: A specific look at 1H-LF-NMR results interpretation. Food Hydrocoll..

[23] Mi H., Tan M., Li J., Li X., Chen J. (2025). Effect of linseed oil and oleogels on the thermal aggregation behavior of myofibrillar protein from Nemipterus virgatus. Food Chem..

[24] Kim S., Jo K., Woo M., Jeong S.-K.-C., Jeon H., Choi Y.-S., Jung S., Lee S. (2025). Interaction mechanisms of κ-carrageenan, gum Arabic, xanthan gum, and sodium alginate with pork myofibrillar proteins: Impacts on heat-induced aggregation and in vitro digestive behaviors of proteins. Food Hydrocoll..

[25] Cao C., Liang X., Xu Y., Kong B., Sun F., Liu H., Zhang H., Liu Q., Wang H. (2024). Effects and mechanisms of different κ-carrageenan incorporation forms and ionic strength on the physicochemical and gelling properties of myofibrillar protein. Int. J. Biol. Macromol..

[26] Chai J., Xu X., Zhao X. (2025). Effects of polysaccharides on the solubilization of myofibrillar protein in aqueous solution: A comparative study. Food Hydrocoll..

[27] Chen J., Wang S., Jia B., Ji Z., Li X., Liu D., Wu Z. (2025). Improvement of gel quality and emulsion stability of high-dose gallic acid-modified myofibrillar protein by zein nanosystems: Emphasizing the role of anionic polysaccharides. Food Chem..

[28] Chang J., Yang X., Li J., Fu Q., Zhou J., Zhao J., Zhang N., Liu Q., Wang T., Wang H. (2023). Improvement of physicochemical and gel properties of chlorogenic acid-modified oxidized myofibrillar proteins by transglutaminase. LWT.

[29] Dadou S.M., El-Barghouthi M.I., Alabdallah S.K., Badwan A.A., Antonijevic M.D., Chowdhry B.Z. (2017). Effect of Protonation State and N-Acetylation of Chitosan on Its Interaction with Xanthan Gum: A Molecular Dynamics Simulation Study. Mar. Drugs.

[30] Majid N., Khan R.H. (2023). Protein aggregation: Consequences, mechanism, characterization and inhibitory strategies. Int. J. Biol. Macromol..

[31] Wang X., Han R., Ramaswamy H., Wang C., Duan H. (2025). Effects of high-pressure processing on physicochemical, structural and emulsifying properties of chicken myofibrillar proteins. LWT.

[32] Wang S., Zhang Y., Chen L., Xu X., Zhou G., Li Z., Feng X. (2018). Dose-dependent effects of rosmarinic acid on formation of oxidatively stressed myofibrillar protein emulsion gel at different NaCl concentrations. Food Chem..

[33] Yang D., Zhang L., Gao S., Luo Y., Luo R., Hou Y. (2025). Effects of grape seed proanthocyanidin on the conformation and functional properties of lamb myofibrillar protein under hydroxyl radical-induced oxidative stress. LWT.

[34] Huang M., Xu Y., Chen X., Xu L., Bai Y., Xu X., Zeng X. (2024). Improved emulsifying properties of water-soluble myofibrillar proteins at acidic pH conditions: Emphasizing pH-regulated electrostatic interactions with chitosan. Int. J. Biol. Macromol..

[35] Bao Y., Boeren S., Ertbjerg P. (2018). Myofibrillar protein oxidation affects filament charges, aggregation and water-holding. Meat Sci..

[36] Huang M., Xu Y., Xu L., Bai Y., Zeng X., Zheng R., Xu X. (2023). Conformation changes and emulsifying properties of myofibrillar proteins in water: Effects of electrostatic interaction with chitosan. Food Res. Int..

[37] Niu H., Wang W., Dou Z., Chen X., Chen X., Chen H., Fu X. (2023). Multiscale combined techniques for evaluating emulsion stability: A critical review. Adv. Colloid Interface Sci..

[38] Zhang Z., Xiao S., Hu Y., Meng X., He X., Bai W., Liu Q. (2025). Chlorogenic acid-Myofibrillar protein interactions: Mechanism and impact on pickering emulsion. Int. J. Biol. Macromol..

[39] Diao X., Wang Y., Jia R., Chen X., Liu G., Liu D., Guan H. (2024). Influences of ultrasonic treatment on the physicochemical properties and microstructure of diacylglycerol-loaded emulsion stabilized with soybean protein isolate and sodium alginate. Ultrason. Sonochem..

[40] Zhao Y., Xu X., Zhao X. (2026). Mechanisms underlying the regulation of oil/water interface behavior by interfacial distribution of myofibrillar proteins and chitosan. Food Hydrocoll..

[41] Wang K., Li Y., Sun J., Zhang Y. (2023). The physicochemical properties and stability of myofibrillar protein oil-in-water emulsions as affected by the structure of sugar. Food Chem. X.

[42] Jiang S., Mo F., Liu Q., Jiang L. (2024). Insights into the in vitro digestibility and rheology properties of myofibrillar protein with different incorporation types of curdlan. Food Chem..

[43] Cui Q., Ya X., Guo C., Yi J., Zhang H., Xu X., Ma Y. (2026). Molecular interaction mechanism between tamarind seed polysaccharide and gluten protein: Insights into structural modulation and physicochemical properties. Int. J. Biol. Macromol..

[44] Wang X., Xia M., Zhou Y., Wang L., Feng X., Yang K., Ma J., Li Z., Wang L., Sun W. (2020). Gel properties of myofibrillar proteins heated at different heating rates under a low-frequency magnetic field. Food Chem..

[45] Tarahi M., Gharagozlou M., Niakousari M., Hedayati S. (2024). Protein–Chlorogenic Acid Interactions: Mechanisms, Characteristics, and Potential Food Applications. Antioxidants.

[46] Yao W., Zhao Z., Zhang J., Kong B., Sun F., Liu Q., Cao C. (2024). Revealing the deterioration mechanism in gelling properties of pork myofibrillar protein gel induced by high-temperature treatments: Perspective on the protein aggregation and conformation. Meat Sci..

[47] Ma Y., Shen R., Wang Y., Wang X., Tian X., Wang W. (2025). Mechanical properties of myofibrillar protein-cellulose nanofibril composite gels: Role of protein concentration and network structure. Food Hydrocoll..

[48] Jiang S., Huang Y., Li Q., Luo T., Guan T. (2025). Effects of three polyphenols on the gel properties and structures of goose myofibrillar protein. Food Hydrocoll..

[49] Szmańko T., Lesiów T., Górecka J. (2021). The water-holding capacity of meat: A reference analytical method. Food Chem..

[50] Zhang Y., Bai G., Wang J., Wang Y., Jin G., Teng W., Geng F., Cao J. (2023). Myofibrillar protein denaturation/oxidation in freezing-thawing impair the heat-induced gelation: Mechanisms and control technologies. Trends Food Sci. Technol..

[51] Nawaz A., Luo X., Irshad S., Dong Z., Li Z., Qin Z., Li C., Khan M.R., Wahab R., Walayat N. (2025). Effect of polyphenol-hydrocolloids interaction on protein oxidation, structure and water distribution properties of thermally processed meat. Food Hydrocoll..

